# Breast cancer in West Africa: molecular analysis of *BRCA* genes in early-onset breast cancer patients in Burkina Faso

**DOI:** 10.1186/s40246-021-00365-w

**Published:** 2021-10-30

**Authors:** Michela Biancolella, Nabonswindé Lamoussa Marie Ouédraogo, Nayi Zongo, Théodora Mahoukèdè Zohoncon, Barbara Testa, Barbara Rizzacasa, Andrea Latini, Chiara Conte, Tégwindé Rebeca Compaore, Charlemagne Marie Rayang-Newendé Ouedraogo, Si Simon Traore, Jacques Simpore, Giuseppe Novelli

**Affiliations:** 1 Department of Biology, University of Rome “Tor Vergata”, 00133 Rome, Italy; 2 Medical Genetics Laboratory, Tor Vergata Hospital, Rome, Italy; 3 University Saint Thomas d’Aquin of Ouagadougou, Saint Camille Hospital, 06 BP: 10212, Ouagadougou 06, Burkina Faso; 4 Research Centre (CERBA), P.O. Box 364, Ouagadougou 01, Burkina Faso; 5 Department of Visceral Surgery of Yalgado, Ouédraogo University Hospital (CHUYO), Joseph KI ZERBO University of Ouagadougou, Ouagadougou, Burkina Faso; 6 Department of Biomedicine and Prevention, University of Rome “Tor Vergata”, 00133 Rome, Italy; 7 Department of the Gynecology of Bogodogo University Hospital, Joseph KI ZERBO University of Ouagadougou, 04 BP 8201, Ouagadougou 04, Burkina Faso; 8 IRCCS Neuromed, Pozzilli, IS Italy; 9 Department of Pharmacology, School of Medicine, University of Nevada, Reno, NV 89557 USA

**Keywords:** Breast cancer, BRCA1, BRCA2, Burkina Faso, West Africa, NGS

## Abstract

**Background:**

Breast cancer (BC) is the most commonly diagnosed cancer and the second leading cause of cancer-related deaths among women in Africa after cervical cancer. Even if the epidemiological data are now aligned with those relating to industrialized countries, the knowledge concerning breast cancer in Africa, particularly in Western Africa, still lack clinical data, medical treatments, and the evaluation of genetic and non-genetic factors implicated in the etiology of the disease. The early onset and the aggressiveness of diagnosed breast cancers in patients of African ancestry strongly suggest that the genetic risk factor may be a key component, but so far, very few studies on the impact of germ line mutations in breast cancer in Africa have been conducted, with negative consequences on prevention, awareness and patient management. Through Next Generation sequencing (NGS), we analyzed all of the coding regions and the exon–intron junctions of *BRCA1* and *BRCA2* genes—the two most important genes in hereditary breast cancer—in fifty-one women from Burkina Faso with early onset of breast cancer with or without a family history.

**Results:**

We identified six different pathogenic mutations (three in *BRCA1*, three in *BRCA2*), two of which were recurrent in eight unrelated women. Furthermore, we identified, in four other patients, two variants of uncertain clinical significance (VUS) and two variants never previously described in literature, although one of them is present in the dbSNP database.

**Conclusions:**

This is the first study in which the entire coding sequence of *BRCA* genes has been analyzed through Next Generation Sequencing in Burkinabe young women with breast cancer. Our data support the importance of genetic risk factors in the etiology of breast cancer in this population and suggest the necessity to improve the genetic cancer risk assessment. Furthermore, the identification of the most frequent mutations of *BRCA1* and *BRCA2* in the population of Burkina Faso will allow the development of an inexpensive genetic test for the identification of subjects at high genetic cancer risk, which could be used to design personalized therapeutic protocols.

**Supplementary Information:**

The online version contains supplementary material available at 10.1186/s40246-021-00365-w.

## Background

The global rate of new cancer cases is increasing worldwide [1]. In 2018, the global cancer observatory estimated that, by 2030, 24 million people worldwide will develop cancer, and 13 million people will die annually from cancer, with 75% of these deaths in low- and middle-income countries [2, 3].

In these countries, the global trend in the epidemiology of breast cancer aligns with that of high-income countries [4]. Focusing on Africa, and specifically on Western Africa, which includes Burkina Faso, breast cancer represents the second most frequent diagnosed cancer in women [4, 5]. However, the lack of resources for preventative screening, as well as access to quality health care, create significant delays in breast cancer detection, contributing to a high mortality rate [6, 7]. Moreover, the genetics of breast cancer in African countries is generally uncertain [8] and although the onset at a young age and the aggressiveness of this breast cancer suggest that there may be a strong inheritance/familial component in the onset of the disease, very few studies have been conducted to address this issue [8–11].

In order to better assess the genetic risk to develop cancer in this population, we analyzed the coding regions and the exon–intron junctions of the two main breast cancer susceptibility genes [12], *BRCA1* [13] and *BRCA2* [14] through next generation sequencing (NGS) in 51 Burkinabe women affected by breast cancer.

To the best of our knowledge, this is the first work that analyzed, with an NGS approach, all the coding sequence of *BRCA1* and *BRCA2* genes in Burkinabe women with breast cancer. The identification and characterization of the most recurrent mutations in these patients will allow the development of genetic tests based on a population-specific mutation panel, which will lower the costs of genetic testing, thus creating the possibility of carrying out preventative screenings in high-risk populations.

Moreover, the early identification of women who carry pathogenic or likely pathogenic germline variants in *BRCA1* or *BRCA2* genes will allow the setup of an appropriate diagnostic-therapeutic path to improve the overall survival rate of breast cancer patients.

## Results

A total of 51 African women from Burkina Faso affected by breast cancer have been selected by genetic counseling at the CERBA/LABIOGENE laboratory of the University of Ouagadougou (Burkina Faso) to perform a genetic testing for the screening of *BRCA1* and *BRCA2* genes. Among the analyzed 51 women, 22 were Mossi (the largest ethnic group in Burkina Faso). The others ethnic groups were 11 Dioula, 6 Bissa, 4 Samo, 3 Gourmatché, 2 Peulh and 3 Gourounsi. The molecular analyses were carried out at the Medical Genetics Laboratory of the University of Rome Tor Vergata, Italy.

All patients were under 40 at the time of their diagnosis. The mean age of the patients at diagnosis was 34.8 ± 4.14. Ninety-four percent (94%) of patients had an invasive ductal type of breast carcinoma, and only 24% (12/51) reported to have family history of breast cancer. It is reported that 3/51 patients have family history of ovarian cancer. Tumors T3 and T4 represented, respectively, 20.7% and 38.0% (p value = 0.041) and had an inflammatory appearance in 34.5% of cases. The palpated axillary lymph nodes were motile in 37 patients (63.8% of cases) and fixed in 15.5% of cases. The analysis of the hormone receptors (ER-PR) status was performed in 45/51 cases. The result was (ER+ = 11/45; ER− = 34/45; PR+ = 16/45; PR− = 29/45). The HER2 status was checked in 28/51 cases. The result was (HER2+ = 11/28; HER− = 17/28).

The NGS analysis produced an output of 644 entries containing the variant code, chromosomal, coding and amino acid position. Of all the entries, 30 were detected only one time (unique variants) (List of the 30 unique variants identified in our cohort are reported in Additional file 1: Table S1). Most of the 30 variants were benign (22); four variants were pathogenic, two were uncertain significance (VUS), two were novel variants.

**Fig. 1:**
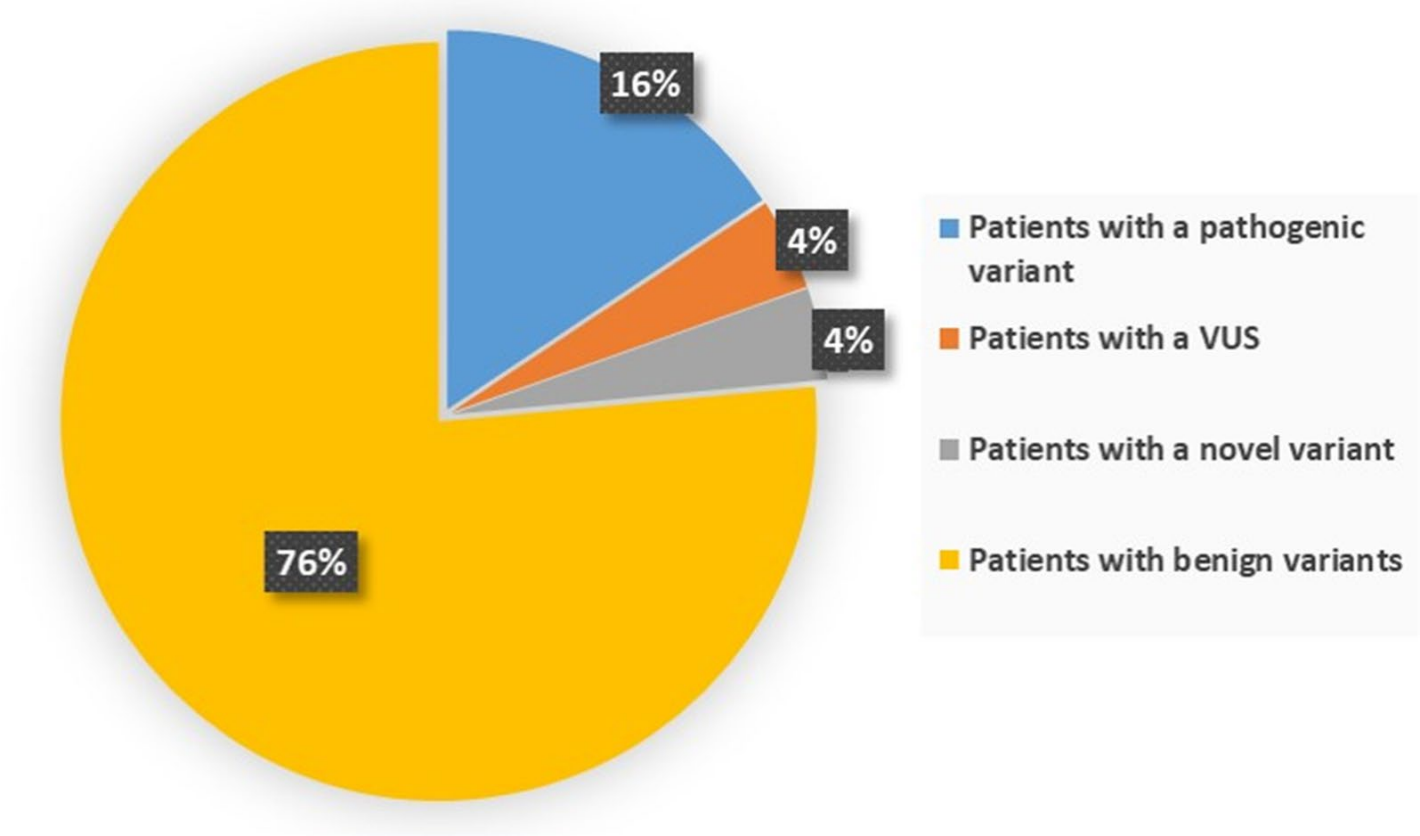
Percentage of patients carrying a pathogenic variant (16%), a VUS (4%), a novel variant (4%) or benign variants (76%)

Overall, on the total of 51 patients analyzed, we found eight carriers of a pathogenic variant (16%), two carriers of a variant of uncertain significance (VUS) (4%) and two carriers of a new undescribed variant (4%) (Fig. 1).

The clinical characteristics of the patients with the identified variants (pathogenetic variants, VUS variants, and novel variants) are shown in Additional file 2: Table S2.

No linkage disequilibrium (LD) was detected between traditional mutations and the new discovered variants. We observed that the median age of patients carrying pathogenic variants was lower (33.25 ± 3.77) compared to the median age of patients carrying only benign variants (35.16 ± 4.16), although this difference was not statistically significant (*p* value = 0.25).

### Pathogenic variants

The identified pathogenic variants were in total six (four as unique variants and two as variants detected more than one time) (Table 1). Three variants were in the *BRCA1* gene and three in the *BRCA2* gene. The *BRCA1* mutation carriers had a mean age of (32.4 ± 3.78); *BRCA2* mutations carriers had a mean age of (34.67 ± 4.04). The mutation prevalence was evaluated in our cohort and resulted to be 15.7% (95% CI: 5.7–25.7%) for the two *BRCA* genes, in particular 9.8% (95% CI: 1.6–18.0%) for *BRCA1* and 5.9% (95% CI: 0.2–11.4%) for *BRCA2.* The mutations included missense, nonsense, small deletion and intronic variants (Table 1).

**Table 1:** Pathogenic variants VUS identified in our study

Gene	Location	HGSV nucleotide	HGSV protein	Number of carriers	dbSNP	Allele frequency	Allele frequency in Africa (gnomAD)	*p* value	All populations Frequency	ACMG criteria
Pathogenic variants										
*BRCA1*	Exonic	c.5177_5180delGAAA	p.Arg1726Lysfs*3	1	rs80357975	0.98%	0.012%	**0.01**	0.0008%	PVS1, PP5, PM2, PM3
	Exonic	c.4088C>G	p.Ser1363*	2	rs398122680	1.96%	0.006%	**0.00003**	0.0004%	PVS1, PP5, PM2, PM3
	Intronic	c.4986+6T>C	p.(?)	2	rs80358086	1.96%	0%	**0.00001**	0.0004%	PP5, PM2, BP4
*BRCA2*	Exonic	c.6445_6446delAT	p.Ile2149*	1	rs80359592	0.98%	Not Reported	-	-	PVS1, PP5, PM2
	Exonic	c.8009C>T	p.Ser2670Leu	1	rs80359035	0.98%	Not Reported	-	-	PP5, PM2, PM3
	Exonic	c.6757_6758delCT	p.Leu2253Phefs*7	1	rs80359623	0.98%	0%	**0.003**	0.0004%	PVS1, PP5, PM2
VUS										
*BRCA1*	Exonic	c.5348T>C	p.Met1783Thr	1	rs55808233	0.98%	0.17%	0.16	0.016%	PM1, PM2, PP3,PP5
*BRCA2*	Exonic	c.7504C>T	p.Arg2502Cys	1	rs55716624	0.98%	0.32%	0.28	0.033%	PM2, BP4

Significant differences are reported in bold

ACMG: American College of Medical Genetics

In particular, in *BRCA1* gene, we identified three pathogenic variants in five unrelated patients. Two patients were affected by the same mutation c.4088C>G, (p.Ser1363*). This is a nonsense mutation which causes the substitution at amino acid 1363 from Serin to a stop codon [15]. Patients carrying this mutation had an undifferentiated and ductal type of breast carcinoma at the ages of 37 and 34, respectively, and both had a family history of breast cancer. Two other patients shared the intronic mutation c.4986+6T>C (LRG_292t1:c.4986+6T>C). This variant has a severe impact on splicing, because it leads to the activation of a downstream cryptic splice donor site, which results in an aberrant RNA transcript and a truncated protein [16]. These patients were affected by a breast ductal carcinoma, diagnosed at early ages of 28 and 29, respectively. One patient reported to have a family history of cancer, while the other patient did not.

Moreover, in another patient, we identified the frameshift mutation, c.5177_5180del, (p.Arg1726Lysfs*3) [17]._._ The patient carrying this mutation had a breast ductal carcinoma, diagnosed at age of 34 and no family history of breast cancer.

In *BRCA2* gene, we identified three pathogenic variants, two frameshift mutations and one missense mutation, in three unrelated patients. The frameshift variant c.6445_6446del, (p.Ile2149*) [18] was identified in a patient with breast ductal carcinoma, diagnosed at age of 37 and no family history of breast cancer; the frameshift variant c.6757_6758del, (p.Leu2253Phefs*7) [15] was identified in a patient with breast ductal carcinoma, diagnosed at age of 37 and with a family history of breast cancer. The missense mutation, c.8009C>T, (p. Ser2670Leu) [19], was present in a 37-years-old woman with a medullary breast carcinoma and a family history for breast cancer.

Interestingly, the pathogenic variants frequency detected in our cohort is statistically different compared to the frequencies listed in the GnomAD database for the African population (*p* value < 0.05), with the two variants -the c.6445_6446del, (p.Ile2149*) and the c.8009C>T, (p.Ser2670Leu)- not even present in the GnomAD database (Table 1).

### Variants of uncertain significance (VUS)

**Fig. 2:**
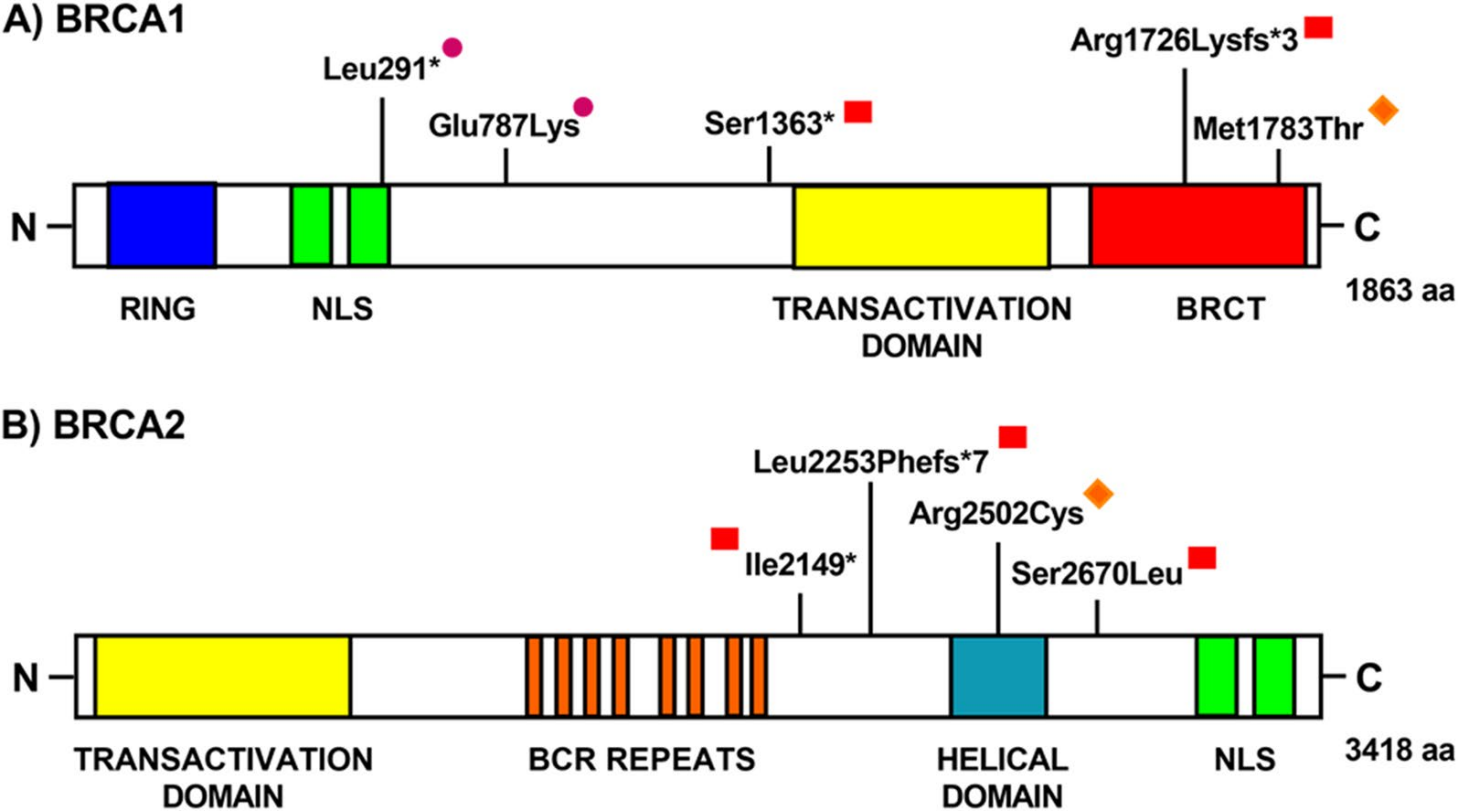
Schematic structure of BRCA1 and BRAC2 proteins showing the identified variants. **A** Schematic structure of BRCA1. RING domain, Nuclear Localization Signals (NLS), Transactivation domain and BRCT domain are indicated. **B** Schematic structure of BRCA2. Transactivation domain, BCR repeats, Helical domain and NLS are indicated. Red square = likely pathogenic/pathogenic variants;, orange rhombus = variants of uncertain significance; purple dot = novel variants

We identified two missense variants one in the *BRCA1* gene (c.5348T>C, p.Met1783Thr) [20] and one in the *BRCA2* gene (c.7504C>T, p.Arg2502Cys) [20] with an allelic frequency not statistically different compared to the frequencies listed in the GnomAD database for the African population (*p* value > 0.05) (Table 1). Both these variants are in a functional domain of the BRCA1 and BRCA2 protein, respectively (Fig. 2).

The variant Met1783Thr is in the BRCT2 domain of BRCA1, while the variant Arg2502Cys is in the BRCA2 helical domain. To predict the potential impact of these variants on the protein, we used different tools (Mutation Taster and PolyPhen-22). The in silico analysis predicted a damaging role for the BRCA1 variant (Mutation Taster: disease causing; PolyPhen-2: Probably damaging, with a score of 1.000); moreover, the sequence alignment of the BRCA1 protein with its orthologous proteins showed that the wild-type residue seemed to be moderately preserved in species, implying a role for this residue in the protein function (Table 2). The in silico analyses for the BRCA2 variant Arg2502Cys gave a benign computational effect on the protein (Mutation Taster: polymorphism; PolyPhen-2: benign, with a score of 0.022). In this case, the sequence alignment of BRCA2 protein with its orthologous proteins showed that the wild-type residue is poorly preserved among species, implying an irrelevant functional or structural role of this residue on the protein.

**Table 2:** Sequence alignment of the BRCA1 and BRCA2 proteins for the two missense VUS identified

SPECIES	MATCH	GENE	AA	ALIGNMENT																						
*BRCA1 c.5348T*>*C (p.Met1783Thr)*																										
Human			1783	T	N	M	P	T	D	Q	L	E	W	**M**	V	Q	L	C	G	A	S	V	V	K	E	L
mutated	Not conserved		1783	T	N	M	P	T	D	Q	L	E	W	**T**	V	Q	L	C	G	A	S	V	V	K	E	
P. troglodytes	All identical	ENSPTRG00000009236	1783	T	N	M	P	T	D	Q	L	E	W	**M**	V	Q	L	C	G	A	S	V	V	K	E	
M. mulatta	All identical	ENSMMUG00000001329	1783	T	N	M	P	T	D	Q	L	E	W	**M**	V	Q	L	C	G	A	S	V	V	K	E	
F. catus	No homologue																									
M. musculus	All identical	ENSMUSG00000017146	1726	T	N	M	P	K	D	E	L	E	R	**M**	L	Q	L	C	G	A	S	V	V	K	E	
G. gallus	All conserved	ENSGALG00000002781	1674	T	D	M	T	T	G	H	L	E	W	**I**	V	E	L	C	G	A	S	V	V	K	Q	
T. rubripes	All identical	ENSTRUG00000009091	1207	T	D	M	T	T	A	E	M	E	L	**M**	V	E	L	C	G	A	T	V	V	K	D	
D. rerio	no homologue																									
D. melanogaster	no homologue																									
C. elegans	No homologue																									
X. tropicalis	All identical	ENSXETG00000024564	1524	T	D	M	T	L	D	D	L	E	W	**M**	V	S	E	C	G	S	T	V	V	R	D	
*BRCA2 c.7504C*>*T (p.Arg2502Cys)*																										
Human			2502	D	M	R	I	K	K	K	Q	R	Q	**R**	V	F	P	Q	P	G	S	L	Y	L	A	
mutated	Not conserved		2502	D	M	R	I	K	K	K	Q	R	Q	**C**	V	F	P	Q	P	G	S	L	Y	L	A	
P. troglodytes	All identical	ENSPTRG00000005766	2502	D	M	R	I	K	K	K	Q	R	Q	**R**	V	F	P	Q	P	G	S	L	Y	L	A	
M. mulatta	no homologue																									
F. catus	No homologue																									
M. musculus	Not conserved	ENSMUSG00000041147	2423	D	M	R	I	K	N	K	E	R	R	**H**	L	R	L	Q	P	G	S	L	Y	L	T	
G. gallus	Not conserved	ENSGALG00000017073	2457	E	M	R	I	K	K	K	Y	R	Q	**N**	I	S	P									
T. rubripes	No homologue																									
D. rerio	not conserved	ENSDARG00000079015	2046	D	M	R	L	R	K	K	K	R	Q	**T**	I	R	P	V	P	G	S	L	Y	L	A	
D. melanogaster	No homologue																									
C. elegans	No homologue																									
X. tropicalis	All conserved	ENSXETG00000017011	2293	E	M	R	I	R	K	K	L	R	Q	**K**	I	K	P	H	P	G	S	L	Y	R	L	

### Novel variants

We identified two unclassified variants (4%) in the *BRCA1* gene: one missense variant, c.2359G>A, (p.Glu787Lys) (rs1288796003) and one nonsense variant, c.872T>A, (p.Leu291*) (Table 3). We used in silico tools (Mutation Taster and PolyPhen-22) to evaluate the potential impact of these variants on the BRCA1 protein.

**Table 3:** Novel variants identified in this study

	Gene	Location	HGSV nucleotide	HGSV protein	Number of carriers	Functional domain	Mutation taster	PolyPhen-2	CADD Score	ACMG Criteria
Novel variants	*BRCA1*	Exonic	c.872T>A	p.Leu291*	1	No	Disease causing	–	35	PVS1, PM2, PP3
		Exonic	c.2359G>A	p.Glu787Lys	1	No	Polymorphism	Possibly damaging	14.77	PM2, BP4

ACMG: American College of Medical Genetics; CADD: Combined Annotation Dependent Depletion

The missense variant c.2359G>A causes a non-conservative amino acid change, the substitution of Glutamic Acid 787 to Lysine. This variant is present on the dbSNP database (rs1288796003), but is not present in any other clinical database (ClinVar, LOVD) and is not reported on GnomAD. The in silico analysis with different software gave conflicting verdicts.

The variant c.872T>A, (p.Leu291*) causes the changing of amino acid 291 from Leucine to stop codon thus breeding a truncated protein that comes shorter of 6916 amino acids. In accordance with the American College of Medical Genetics (ACMG), this variant is classified likely pathogenic-class 4 [21].

## Discussion

In Africa and Sub-Saharan Africa (SSA), breast cancer (BC) is the most diagnosed cancer in women, with an increasing incidence in the last few years, and a survival rate five years lower that of industrialized countries [22]. Epidemiological studies would be very useful to better identify risk factors and understand the genetic variability among African populations. However, so far, data are scarce, which significantly impacts the accuracy of diagnosis and clinical management of patients with African ancestry.

The prevalence of mutations in *BRCA* genes is not yet well defined in Western Africa. Data are scarce for most countries and the results of the few studies carried out do not allow to have a real picture of the specific mutations in each country. The prevalence and spectrum of germline mutations in *BRCA1* and *BRCA2* genes are certainly better delineated in European and North American populations [15]. For many of these populations, recurrent and founder mutations have been identified and this has allowed the development of targeted and affordable genetic tests, which can be more easily used for population screening [23]. One example is represented by the Ashkenazi Jewish population in which identified founder mutations have long been used as the first genetic screening test for women of Jewish descent [24]. Founder mutations have also been identified in different European and Asian populations, while for West Africa, only one mutation in the *BRCA1* gene has been identified as a potential founder mutation [25]. These examples suggest that specific mutation panels can be developed for specific population, therefore, it is important for Africa to identify and characterize the recurrent mutations [11].

In this study, we determined the prevalence of mutations in *BRCA* genes in a cohort of young Burkinabe women with breast cancer. Interestingly, the frequency of all identified pathogenic variants, some of which are present in more than one patient, was statistically different from that one reported in the GnomAD database for the African population, with two variants that were not even present in the database. This result suggests that the identified variants could be considered as population-specific variants and therefore be extremely important for genetic testing strategies. In addition to the pathogenic variants, we have identified two variants of uncertain clinical significance (VUS) and two variants never described in the literature only one of which had been previously reported in the dbSNP database alone. The two VUS are in functional domains, and the in silico analysis has predicted a damaging role for the VUS in the *BRCA1* gene and a benign computational effect on the protein for the VUS in the *BRCA2 g*ene. Both variants have an allelic frequency not statistically different to the one listed in the GnomAD database for the African population suggesting their unlikely contribution, on their own, to the risk of developing cancer. However, the role of these variants should certainly be better investigated using multifactorial models and functional studies [26]. To overcome difficulties in classifying *BRCA1 /2* VUS, the Evidence-Based Network for the Interpretation of Germline Mutant Alleles (ENIGMA) consortium was born in 2009 [27]. The International Agency for Research on Cancer Working Group (IARC), in collaboration with ENIGMA, has developed a five-level multifactorial model to classify VUS identified in *BRCA* genes, based on the segregation of the variant in families, the co-occurrence with previously identified pathogenic mutations and tumor histopathology, combined with an analysis of the sequence conservation and properties of mutated residues [28].

In our study, we could not carry out the segregation analysis as we did not have the DNA of others family members nor functional studies; only the in silico analysis has been performed.

The two novel variants identified were in the *BRCA1* gene. One is a nonsense variant that, in accordance with ACMG and ENIGMA criteria, we have classified as likely pathogenic; the other is a missense variant that needs further investigations. Finally, 17 patients (those with better quality DNA) tested negative for *BRCA1/2* mutations were screened for larger genomic rearrangements (LGRs) in BRCA genes by multiplex ligation-molecular-dependent probe amplification (MLPA). No genomic rearrangements were identified in any of the patients analyzed. These data are in agreement with the results obtained by Zhang et al. [29], showing that the genomic rearrangements do not contribute significantly to BRCA-associated risk in the Nigerian population.

This study has limitations: the number of samples analyzed and that the screening only covered the BRCA genes. Furthermore, the sequence study was not extended to the regulatory and intronic regions. Our next step will be to analyze those patients tested negative for BRCA1/2 mutations with a larger gene panel which will include others important breast cancer susceptibility genes in order to have a more complete picture of genetic risk factors in Africa similar to that of European and American countries [30, 31].

## Conclusions

Certainly, the socioeconomic conditions that lead to a weak health care system, the lack of health insurance, limited access to drugs and therapies and a lack of genetic tests have a strong impact on the high mortality and incidence rates of breast cancer in African countries. We believe that our study, although conducted on a limited number of patients, represents an important contribution to add greater knowledge to the genetic risk factors of BC in Western Africa. This will allow the creation of cancer prevention programs and will allow Burkinabe women at high risk of breast cancer to be included in an appropriate diagnostic-therapeutic programs. In fact, it will help reduce the disparities that still exist between being a breast cancer patient in a low-income or a high-income country.

## Methods

### Patients’ recruitment

This was a prospective cohort study which took place from August 1st, 2015 to February 29th, 2016. It consisted of a genetic analysis of breast cancer confirmed cases by histopathological analyses among women younger than 40 years at the University hospital Center of Yalgado OUEDRAOGO (CHU-YO). An approval was obtained from the Ethics Committee for Health Research of Burkina Faso (N° 2014-8-098). After obtaining written informed consent from each patient, clinical, paraclinical and therapeutic data were collected in General Surgery, Gynecology-Obstetrics, Oncology and Anatomy Pathology departments of the CHU-YO. Women younger than 40 years with histologically confirmed breast cancer, attending the latter mentioned departments, who gave their free and written consent to participate, were included in the present study.

The parameters studied for each patient were epidemiological and socio-demographic characteristics (age, sex, occupation, weight, height, level of education, origin); clinical data (antecedents, consultation time, reasons for consultation, symptoms and physical signs). Data collection was possible through interview, physical exanimation, and investigations. For each patient, the tumor was classified according to the cTNM (clinical classification in Tumor-Nodes-Metastasis) and pTNM (p = pathologic) classification of breast cancers (7" edition 2010), and graded according to Scarff-Bloom and Richardson (SBR) grading system.

### DNA extraction and NGS analysis

DNA extraction and NGS analyses were performed at Medical Genetics Laboratory of University of Rome Tor Vergata. Total DNA was isolated from peripheral blood using the QIAGEN® EZ1 DNA Blood 200 μl kit (Qiagen) with the BioRobot EZ1 Workstation (Qiagen, Valencia, CA, USA). The concentration and quality of DNA were determined using NanoDrop 1000 (Thermo Fisher Scientific) and the Qubit Fluorometer 2.0 (Thermo Fisher Scientific). The NGS analyses were performed using Ion AmpliSeq™ BRCA1 and BRCA2 custom Panel (Thermo Fisher Scientific, Inc). The panel consists of three primers pools (55 amplicons) targeting the entire coding region and the exon–intron boundaries of genes BRCA1 and BRCA2. A total of 10 ng of DNA for sample was used for library preparation, using the Ion AmpliSeq™ Library kit 2.0 (Ion Torrent; Thermo Fisher Scientific, Inc.). Each library was barcoded using Ion Xpress™ Barcode Adapters kit (Ion Torrent; Thermo Fisher Scientific, Inc.). After the amplification step follows the emulsion reaction which creates aqueous droplets that randomly trap one or more DNA fragments. Libraries were purified using Agencourt Ampure XP Beads, quantified with the Qubit version 2.0 fluorometer (Thermo Fisher Scientific) using the Qubit dsDNA HS assay kit and diluted approximately 100 pmol/L for PGM while for S5 30 pM. Templated Ion Sphere Particles (ISPs) were loaded into an Ion 510 Chip (Thermo Fisher Scientific) or Ion 316 Chip (Thermo Fisher Scientific). Sequencing was performed on an Ion S5 Platform using the Ion S5 Sequencing kit (Thermo Fisher Scientific) and on Ion PGM Platform using the Ion PGM sequencing kit. All protocols were followed as recommended by the manufacturers without modification.

### Data analysis

Preinstalled plugin in the Torrent Browser generates Binary Alignment/Map (BAM) and variant call format (VCF) files. Raw sequence data were processed using the *Torrent Suite*™ software (Ion Torrent; Thermo Fisher Scientific, Inc.) to analyze barcode reads, to align reads to the HG19 reference genome (Genome Reference Consortium GRCh37) and to generate run metrics, including chip loading efficiency and total read counts and quality. Coverage analysis and variant calling used Torrent Variant Caller plugin software in the Torrent Server*.* We analyzed bam files on IGV (Integrative GenomeViewer) [32] to verify the real coverage of genes and the presence of variants, and on Ion Reporter Thermo Fisher Scientific, Inc.) that allow annotation of single nucleotide variants, insertions, deletions and splice site alterations.

Variants were annotated according to nomenclature used by the Human Variation Society [33]. All the annotations and variants were determined using *BRCA1* (NM_007294.3) and *BRCA2 (*NM_000059.3) as reference transcripts. All candidate variants were required on both sequenced DNA strands with a minimum depth of 50X.

### Variant classification

All the detected sequence variations were submitted to following databases: BRCA Exchange [34], ClinVar database [35], Leiden Open Variation Database (LOVD) [36], and compared with literature data.

The variants were classified using the guidelines providing by the American College of Medical Genetics and Genomics (ACMG) [37].

The in silico analysis to predict the potential impact of the variants on the structure and function of the protein was performed using the following tools: PolyPhen2 [38], and Mutation Taster [39]. The evaluation of the novel variants has been based on the location, type and evolutionary conservation of mutated amino acids, biophysical and biochemical differences between wild-type and mutant amino acid, the in silico analysis of the mutant sequence protein.

### Results’ validation by Sanger sequencing

All the variants of class 3 (variants uncertain significance) class 4/5 (likely pathogenic/pathogenic) and the novel variants detected by NGS were confirmed by bidirectional Sanger sequencing.

The sequencing was performed using the *BigDye Terminator v3.1 Cycle Sequencing kit* (Applied Biosystems; Thermo Fisher Scientific, Inc.) and the *ABI 3130xl Automated Sequencer* (Applied Biosystems, Foster City, CA, USA). The results were analyzed using *Sequencing Analysis 5.2.0* software.

### Multiplex ligation-dependent PCR amplification (MLPA)

The presence of large genomic rearrangements (LGRs) in BRCA1 and BRCA2 gene was investigated by multiplex ligation-dependent probe amplification (MLPA) assay using MLPA commercial kits from MRC Holland (Multiplex Ligation-dependent Probe Amplification, BRCA1: P002, BRCA2: P045) according to the manufacturer’s instructions. We used 100 ng of DNA from each sample, three reference samples and tests were performed in duplicate in the same experiment. The procedures were performed according to the manufacturer’s instructions.. The analysis of fragments was performed on ABI 3130xl sequencer and the data generated were imported and analyzed in Coffalyser Net Software (v.140721.1958).

### Statistical analysis

In this study, the analyses focused only on mutations that are classified as pathogenic. We calculated the mutation prevalence and exact 95% confidence interval (CI) using a Binomial distribution. Differences between alleles frequencies of our examined cohort and those listed in the GnomAD database [40] for the African population were evaluated by Fisher’s exact test. *p* values less than 0.05 were considered statistically significant.

## Supplementary Information

**Additional file 1.** **Table S1.** List of the 30 unique variants identified in our cohort.

**Additional file 2.** **Table S2.** Clinical characteristics of the 12 patients carrier of pathogenic, VUS and novel variants.

## Data Availability

All data generated or analyzed during this study are included in this published article.
