# Supplementary material for: Breast cancer in West Africa: molecular analysis of BRCA genes in early-onset breast cancer patients in Burkina Faso
Source: Hum Genomics. 2021 Oct 30;15:65. doi: 10.1186/s40246-021-00365-w (PMC8557567; doi:10.1186/s40246-021-00365-w)
Supplement: Supplementary file 1 — Additional file 1. Table S1. List of the 30 unique variants identified in our cohort. [file 40246_2021_365_MOESM1_ESM.docx]

**Table S1**. List of the 30 unique variants identified in our cohort.

| **Gene** | **Location** | **HGSV nucleotide** | **HGSV protein** | **dbSNP** |
| --- | --- | --- | --- | --- |
| *BRCA1* | Exonic | c.21C>T | p.Arg7= | rs149402012 |
|  | Intronic | c.135-42G>C | p.(?) | rs377388626 |
|  | Intronic | c.441+15T>C | p.(?) |  |
|  | Intronic | c.442-34C>T | p.(?) | rs799923 |
|  | Intronic | c.547+64G>C | p.(?) | rs144033340 |
|  | Exonic | c.872T>A | p.Leu291* | - |
|  | Exonic | c.1233T>G | p.Asp411Glu | rs80357024 |
|  | Exonic | c.1971A>G | p.Gln657= | rs28897679 |
|  | Exonic | c.2077G>A | p.Asp693Asn | rs4986850 |
|  | Exonic | c.2359G>A | p.Glu787Lys | - |
|  | Exonic | c.2458A>G | p.Lys820Glu | rs56082113 |
|  | Exonic | c.3119G>A | p.Ser1040Asn | rs4986852 |
|  | Exonic | c.3600G>C | p.Gln1200His | rs56214134 |
|  | Intronic | c.4096+36A>G | p.(?) | rs45620639 |
|  | Exonic | c.5177_5180delGAAA | p.Arg1726Lysfs*3 | rs80357975 |
|  | Intronic | [c.5215+27C>A](https://varsome.com/variant/hg19/BRCA1(NM_007300.4):c.5215+27C%3eA?&annotation-mode=germline) | p.(?) |  |
|  | Exonic | c.5348T>C | p.Met1783Thr | rs55808233 |
| *BRCA2* | Exonic | c.231T>G | p.Thr77= | rs114446594 |
|  | Intronic | c.793+65G>T | p.(?) | rs1253006401 |
|  | Exonic | c.1798T>C | p.Tyr600His | rs75419644 |
|  | Exonic | c.3869G>A | p.Cys1290Tyr | rs41293485 |
|  | Exonic | c.5697T>C | p.Asp1899= | rs786203565 |
|  | Exonic | c.6220C>A | p.His2074Asn | rs34309943 |
|  | Exonic | c.6445_6446delAT | p.Ile2149* | rs80359592 |
|  | Exonic | c.6757_6758delCT | p.Leu2253Phefs*7 | rs80359623 |
|  | Intronic | c.7435+6G>A | p.(?) | rs81002852 |
|  | Exonic | c.7504C>T | p.Arg2502Cys | rs55716624 |
|  | Exonic | c.7626G>A | p.Thr2542= | rs61754138 |
|  | Exonic | c.8009C>T | p.Ser2670Leu | rs80359035 |
|  | Intronic | c.8488-32A>G | p.(?) | rs756837938 |
