# Supplementary material for: Breast cancer in West Africa: molecular analysis of BRCA genes in early-onset breast cancer patients in Burkina Faso
Source: Hum Genomics. 2021 Oct 30;15:65. doi: 10.1186/s40246-021-00365-w (PMC8557567; doi:10.1186/s40246-021-00365-w)
Supplement: Supplementary file 2 — Additional file 2. Table S2. Clinical characteristics of the 12 patients carrier of pathogenic, VUS and novel variants. [file 40246_2021_365_MOESM2_ESM.docx]

| **Gene** | **HGVS nucleotide** | **Clinical Significance** | **Age**  **At diagnosis** | **Family history of BC or ovarian cancer** | **Tumor classification (pTNM)** | **Tumor grade**  **(SBR)** | **Histopathological status** | **ER/PR status** | **HER2 status** |
| --- | --- | --- | --- | --- | --- | --- | --- | --- | --- |
| BRCA1 | c.4986+6T>C | Pathogenic | 28 | BC (mother) | pT4N2M1 | Grade III | Invasive ductal carcinoma | ER-, PR- | No exam |
|  |  |  | 29 | Absence | pT2N0M0 | Grade II | Invasive ductal carcinoma | ER-, PR- | HER2+ |
|  | c.5177_5180delGAAA | Pathogenic | 34 | Absence | pT3N1M0 | Grade III | Invasive ductal carcinoma | ER-, PR- | HER2- |
|  | c.4088C>G | Pathogenic | 37 | BC (sister) | pT2N0M0 | Grade II | Invasive ductal carcinoma | ER+, PR- | HER2+ |
|  |  |  | 34 | BC (sister) | pT3N1M1 | Grade II | Invasive ductal carcinoma | ER-, PR- | No exam |
|  | c.5348T>C | VUS | 39 | Absence | pT4N2M1 | Grade III | Invasive ductal carcinoma | ER-, PR- | HER2- |
|  | c.872T>A | Novel | 34 | Absence | pT2N0M0 | Grade II | Invasive ductal carcinoma | ER-, PR- | HER2- |
|  | c.2359G>A | Novel | 33 | Absence | pT2N1M0 | Grade II | Invasive ductal carcinoma | ER-, PR+ | HER2- |
| BRCA2 | c.6445_6446delAT | Pathogenic | 37 | Absence | pT3N1M0 | Grade III | Invasive ductal carcinoma | ER+, PR+ | No exam |
|  | c.8009C>T | Pathogenic | 37 | BC (mother) | pT4N2M1 | Grade III | Medullary carcinoma | ER-, PR- | HER2- |
|  | c.6757_6758delCT | Pathogenic | 37 | BC (mother) | pT4N2M1 | Grade III | Invasive ductal carcinoma | ER-, PR- | HER2- |
|  | c.7504C>T | VUS | 27 | Absence | pT4N2M1 | Grade III | Invasive ductal carcinoma | ER-, PR- | No exam |

***Table S2.*** *Clinical characteristics of the 12 patients carrier of pathogenic, VUS and novel variants*
